# Applications of the Reaction Interface/Mass Spectrometer Technique to the Analysis of Selected Elements and Nuclides from Submicrogram Quantities of Biological Macromolecules and Xenobiotics

**DOI:** 10.6028/jres.093.102

**Published:** 1988-06-01

**Authors:** Donald H. Chace, Fred P. Abramson

**Affiliations:** Department of Pharmacology, George Washington University, School of Medicine, 2300 Eye Street N.W., Washington, DC 20037

Markey and Abramson [1] developed a microwave-powered chemical reaction interface, a device which converts a complex organic molecule in the presence of a reactant gas into small stable molecules which are detected by mass spectrometry. For a given reactant gas the molecules formed are a representation of the elemental composition of the original anlayte. The combination of the reaction interface and a mass spectrometer produces an isotope- or element-selective detector for samples either introduced directly into the reaction interface or flowing from a capillary gas chromatograph column.

Microgram and submicrogram samples of a variety of proteins were analyzed for their sulfur content relative to their carbon content by introducing the samples directly into the reaction interface. With CO, as the reactant gas, SO_2_ at *m*/*z* 64 is produced. This quantifies the amount of sulfur which was introduced into the reaction interface. In the presence of N_2_, HCN at *m*/*z* 27 is produced and is used to quantify the carbon content of the sample. The observed ratio of S/C for various proteins correlated well with the elemental formulas [2].

In the presence of SO_2_, ^14^NO at *m*/*z* 30 and ^15^NO at *m*/*z* 31 are produced. Following administration of 50 mg of triple-labeled 5,5-diphenylhydantoin [1, 3(^15^N); 2(^13^C)] to a male beagle dog, a urine sample was selectively analyzed for its ^15^N content by capillary gas chromatography—reaction interface/mass spectrometry. The corrected ratio of *m*/*z* 31 to *m*/*z* 30 produced a highly selective chromatogram showing only peaks of ^15^N enrichment. Mass spectra of these peaks were obtained which permitted identification of phenytoin and several of its metabolites.
